# A 10-m national-scale map of ground-mounted photovoltaic power stations in China of 2020

**DOI:** 10.1038/s41597-024-02994-x

**Published:** 2024-02-13

**Authors:** Quanlong Feng, Bowen Niu, Yan Ren, Shuai Su, Jiudong Wang, Hongda Shi, Jianyu Yang, Mengyao Han

**Affiliations:** 1 College of Land Science and Technology, China Agricultural University, Beijing, 100193 China; 2 Institute of Geographic Sciences and Natural Resources Research, Chinese Academy of Sciences, Beijing, 100101 China; 3 Centre for Environment, Energy and Natural Resource Governance (C-EENRG), University of Cambridge, Cambridge, CB2 3QZ United Kingdom

**Keywords:** Geography, Energy supply and demand

## Abstract

We provide a remote sensing derived dataset for large-scale ground-mounted photovoltaic (PV) power stations in China of 2020, which has high spatial resolution of 10 meters. The dataset is based on the Google Earth Engine (GEE) cloud computing platform via random forest classifier and active learning strategy. Specifically, ground samples are carefully collected across China via both field survey and visual interpretation. Afterwards, spectral and texture features are calculated from publicly available Sentinel-2 imagery. Meanwhile, topographic features consisting of slope and aspect that are sensitive to PV locations are also included, aiming to construct a multi-dimensional and discriminative feature space. Finally, the trained random forest model is adopted to predict PV power stations of China parallelly on GEE. Technical validation has been carefully performed across China which achieved a satisfactory accuracy over 89%. Above all, as the first publicly released 10-m national-scale distribution dataset of China’s ground-mounted PV power stations, it can provide data references for relevant researchers in fields such as energy, land, remote sensing and environmental sciences.

## Background & Summary

As an indispensable part of renewable energy sources, photovoltaic (PV) power has drawn increasingly more attention around the globe nowadays^1,2^. The total global capacity of PV power has been reached 115 GW in 2019^2^, justifying the significance of PV power in energy industries, especially in the context of fossil fuel shortage and overexploitation. Meanwhile, PV power belongs to an environmentally friendly way for energy utilization with few carbon emissions^3,4^, which shows a positive effect against global warming.

Due to the above advantages, PV power stations were firstly welcomed in developed countries such as the United Kingdom^5^ and the United States^6^. Both large-scale ground-mounted PV power stations and distributed roof-mounted PV panels emerged with great speed. Meanwhile, PV power has gradually raised huge concerns in China. According to statistics^7^, the installed capacity of PV power in China was only 100 MW in 2007, but grew rapidly to 205,000 MW in 2019, with an average growth of 17,075 MW per year. It should be noted that China’s central government released the Carbon Peak and Carbon Neutrality strategy in 2020, which committed that China’s carbon emissions would reach the peak by 2030 and achieve carbon neutrality by 2060^8^. Therefore, it is predictable that PV power would play an increasingly essential role in the near future. The monitoring of PV power stations would be meaningful for both researchers and government officials.

**Fig. 1:**
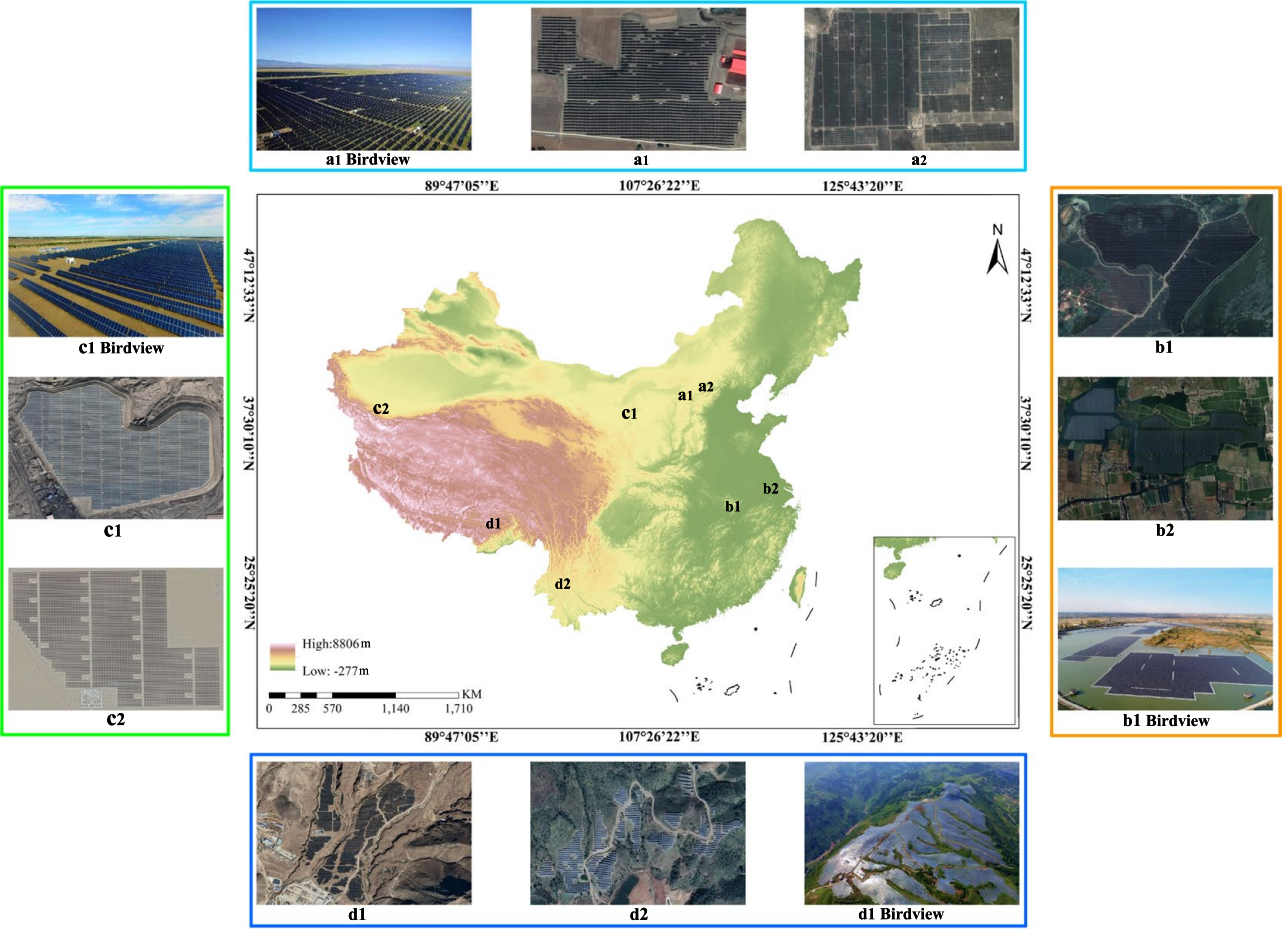
Examples of PV power stations in China. The land used for PV power stations includes gobi (left), grassland (top), water bodies (right), mountain land (bottom), etc.

As mentioned above, the last decade has witnessed the widespread of PV power stations in China, where much previous gobi, grassland, water bodies and mountain land have now been covered by newly-built PV power stations (Fig. 1). When looking into the publicly released scientific data of China’s PV power stations, only the statistical data of PV’s installed capacity for each province could be achieved, lacking the spatial distribution data that could provide more details of China’s PV power industry. Although some researchers released several PV power station maps, most only met a medium resolution of 30 meters^9,10^. There thus still lacks a national map of China’s PV power stations with a higher spatial resolution (i.e., 10 meters) that could provide a global understanding of PV’s spatial deployment patterns. Considering that the large-scale grounded-mounted PV power stations almost cover more than 90% of the total PV capacity in China, we attempt to provide the first publicly available 10-m national map of ground-mounted PV power stations in this dataset.

As for PV power station mapping, previous methods mainly focused on field survey and visual inspection, where manual annotation was performed to delineate the locations or boundaries based on the remote sensing imagery. However, this approach is time-consuming with low efficiency, especially when facing the task of national-scale or continental-scale mapping. To tackle this issue, several researchers resorted to automatic mapping methods such as machine learning^11–13^ or deep learning^14–18^. A general pipeline includes sample selection, feature extraction, model training and validation, accuracy assessment, etc. For instance, Plakman *et al*.^11^ designed an object-based random forest classification method for solar park detection in Netherlands and achieved a user accuracy of 92.39%. Costa *et al*.^15^ utilized several deep semantic segmentation models to map the PV solar plants in Brazil with an IoU (intersection of union) of 91%.

Although deep learning has been popular nowadays, the training of a deep neural network needs a huge number of labelled samples^19^. Otherwise, the deep learning model would be easily overfitted on limited training samples and show poor performance when predicting the new unseen datasets. Under this context, classic machine learning methods such as random forest (RF)^20^ together with powerful cloud computing platforms like Google Earth Engine (GEE, https://code.earthengine.google.com/)^21^ would be a better choice for the task of large-scale mapping from remote sensing data.

The objective of this study is to provide the first publicly released 10-m national map of ground-mounted PV power stations of China in 2020. Specifically, Sentinel-2 multi-spectral imagery^22^ was used as data sources, from which random forest classifier was utilized to predict these PV power stations via GEE cloud computing platform. Both partition modelling and active learning strategy were adopted to tackle the data unbalance problem in large-scale mapping. In addition, the fine-scale classification data of PV power stations could also provide a huge amount of labelled samples, making it possible to train a sophisticated deep learning model in the near future.

Above all, we provide a 10-m national-scale map for PV power stations in China of 2020, which would be of particular interest to the following research areas.

### Estimation and prediction of PV’s generating capacity

Based on the fine-scaled national map of PV power stations, it would be possible to estimate and predict the accurate generating capacity, when considering both solar radiation and weather conditions above these PV power stations^23^.

### Site selection for newly built PV power stations

It would be much easier for the site selection of future PV power stations in China^24,25^ according to the dataset provided in this study.

### Land change

According to previous land use land cover (LULC) data and the PV power station map^26^, it would be interesting to study where, how, and why the other LULC changes into PV power stations.

### Energy policy

The detailed map of PV power stations would provide more clues to test the effect of China’s newly established energy policies^8^.

### Huge and high-quality samples of PV power stations

This dataset could also provide a large number of PV power station samples within China with high quality, which makes it possible to train a robust deep learning model^17^.

## Methods

### Overall workflow

**Fig. 2:**
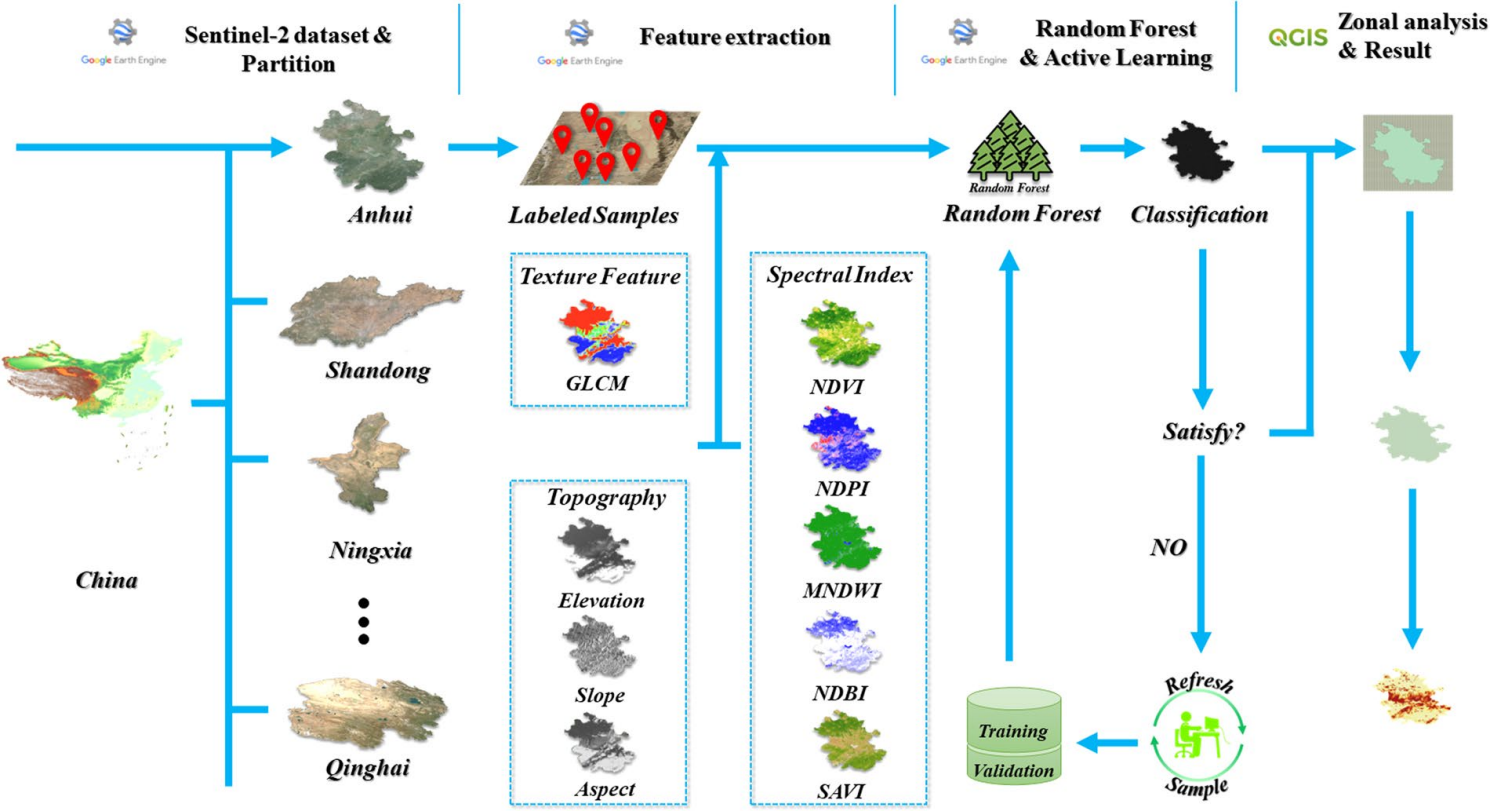
Overall workflow of this study. Both GEE cloud platform and local processing (QGIS) are used. The methods consist of data partition, feature extraction, random forest classification & active learning and zonal analysis.

The overall workflow is depicted in Fig. 2, including study area partition, feature extraction, PV power station classification based on random forest and active learning, post-processing and statistical analysis.

Specifically, when performing national-scale PV power station mapping, if only one classification model was built, it would be difficult to yield high accuracy due to the samples’ distribution variation across large regions. To tackle this issue, we adopt the partition methodology, where a series of RF classifiers have been built for each province of China to increase the model’s sensitivity to local samples. Afterwards, a multi-dimensional feature space could be constructed, which consists of spectral features, texture features and topographical features, aiming to increase the inter-class separability. Next, active learning is synthesized with RF classifier to perform a coarse-to-fine procedure for PV power station mapping. Finally, post-processing is adopted to remove the isolated noises while spatial statistics (i.e., zonal analysis) are performed for subsequent analysis.

### Input data

#### Sentinel-2

As an important part of European Space Agency’s (ESA) Copernicus mission, Sentinel-2 satellite provides a high resolution (10-m) multi-spectral remote sensing data with a global coverage^22^. Compared with NASA’s Landsat data, Sentinel-2 shows two major advantages, making it a better choice for national-scale PV power station mapping. Firstly, Sentinel-2 has a finer spatial resolution (10-m) than that of Landsat (30-m), which could show more details of PV power stations. Besides, the mixed pixel would also be relieved for Sentinel-2 data due to the high resolution, which could increase the quality of classification results, especially at the boundaries. Secondly, Sentinel-2 has a shorter revisit period (5 days) than Landsat (15 days), which makes it easier for GEE to prepare the cloud-free data for subsequent PV power station classification. Since Sentinel-2 consists of two satellites, Sentinel-2A and Sentinel-2B, it could be operated in a collaborative way to shorten the revisit time to provide denser sequential observation data than Landsat. When performing the national-scale or continental-scale classification, the cloud-free remote sensing data is of great significance. These two reasons stated above make Sentinel-2 a better choice for PV power station mapping across China.

Meanwhile, as for the period of Sentinel-2 images, the spring of 2020 was selected from March to May. The reason is as follows. Firstly, winter time was not select because there is a high probability that the snow would cover PV panels, which would lead to classification errors between snow and PV power stations. Summer and autumn were not considered because these two seasons witness a flourishing vegetation. As there are also some vegetation (mostly grass) inside the PV power stations, there would be a high possibility to confuse PV stations and other vegetations. When in spring, most vegetation just turns green, PV power stations and the surroundings show a great difference, making it easier to separate them from each other hence to achieve a high classification accuracy. Besides, due to the existence of clouds, multi-temporal images were used rather than mono-temporal image. The reason is that it is impossible to obtain national-scale cloudless remote sensing images within a single time phase. To tackle this issue, the period from March to May was selected and the image composition method from GEE was used to get the cloudless data.

Moreover, GEE offers an official code for data pre-processing and data composition over large-scale regions. Users just set a starting date and an end date, GEE will automatically stitch the satellite images within the time interval to avoid both data missing and cloud coverage. This official code proves to be effective in large-scale mapping and has been widely used by previous researchers^21^. In this study, we selected the satellite images from March to May of 2020 to shorten the time interval to reduce the possibility of witnessing the inconsistency of satellite images.

#### DEM

Considering that the locations of PV power stations are closely related to terrains, therefore, we also considered the topographic features as input variables for PV power station classification. The reason is simple, as the PV panel should be placed tilted where its normal is coincided with the solar incident angle to get as much solar radiation as possible, most PV power stations would be built on flat ground or sunny side rather than the shady side of the mountain. Therefore, through the inclusion of topographic features such as aspect and slope, it would increase the inter-class separability between PV power stations and other land objects to further improve the mapping performance.

Specifically, the digital terrain data used is ALOS World 3D-30m (AW3D30)^27^ provided by GEE. According to previous study^28^, AW3D30 is the most accurate DEM to date among all the free DEMs with a horizontal resolution of 30-m. In this study, to co-register with Sentinel-2 data, AW3D30 data was resampled to 10-m resolution using a bilinear interpolation method, after which both the slope and aspect were derived.

### Data pre-processing

#### Spectral and texture features derived from Sentinel-2

Feature extraction is of great significance for remote sensing based classification. In this study, a series of spectral and textual features are calculated based on Sentinel-2 multi-spectral data to construct a multi-dimensional feature space, aiming to increase the inter-class differences.

Specifically, the following spectral features are utilized, including normalized difference vegetation index (NDVI), soil-adjusted vegetation index (SAVI), modified normalized difference water index (MNDWI), and normalized difference building index (NDBI). NDVI is used to enhance the differences between vegetation and PV power stations, while SAVI could further correct the NDVI value influenced by soil brightness in areas where the vegetation coverage is rather low. MNDWI is a widely used water index, which could increase the separability of PV power stations and water bodies. NDBI is for the separation of PV power stations and buildings. In addition, a newly proposed spectral index for PV power station recognition, normalized difference PV index (NDPI)^29^, has also been introduced as follows.

1 $$NDPI=\frac{\rho (SWIR1)-\rho (NIR)}{\rho (NIR)-\rho (SWIR2)}$$

where *ρ*(SWIR1) and *ρ*(SWIR2) refer to the two short wave infrared bands of Sentinel-2, i.e., Band-11 and Band-12, respectively, while *ρ*(NIR) represents Sentinel-2’s near infrared band (B8). Since the PV panels show an obvious reflectance peak in Band-11 (SWIR1) and an absorption bottom in both Band-8 (NIR) and Band-12 (SWIR2), the design of NDPI would highlight the PV panels against other land objects.

Meanwhile, compared with other natural land covers such as grassland and bare soil, PV power stations have regular boundaries with clear textures observed from satellite. The rectangle shape of PV power stations would result in different texture features with other land covers, therefore, the inclusion of texture features could improve the classification performance. In this study, the popular gray level co-occurrence matrix (GLCM) was used for texture feature calculation, including mean, variance, homogeneity, dissimilarity, entropy, correlation, inverse difference moment and angular second moment. Besides, the GLCM texture window size is 3 × 3, according to our previous studies^30^. To make the manuscript more readable, a table that contains all the necessary terminologies and their definitions could be found in the Supplementary file (Table S1).

#### Slope and aspect derived from DEM

As stated above, during the site selection of large-scale ground-mounted PV power stations, there is an inclination to choose the flat ground or sunny side of the mountains to receive more solar radiation. Therefore, the inclusion of PV location sensitive features such as slope and aspect would help in differentiating PV power stations from other land covers.

To further justify the above hypothesis, we randomly selected a total of 100, 000 PV samples directly from the final released national PV power station maps to calculate the distribution of both aspect and slope.

**Fig. 3:**
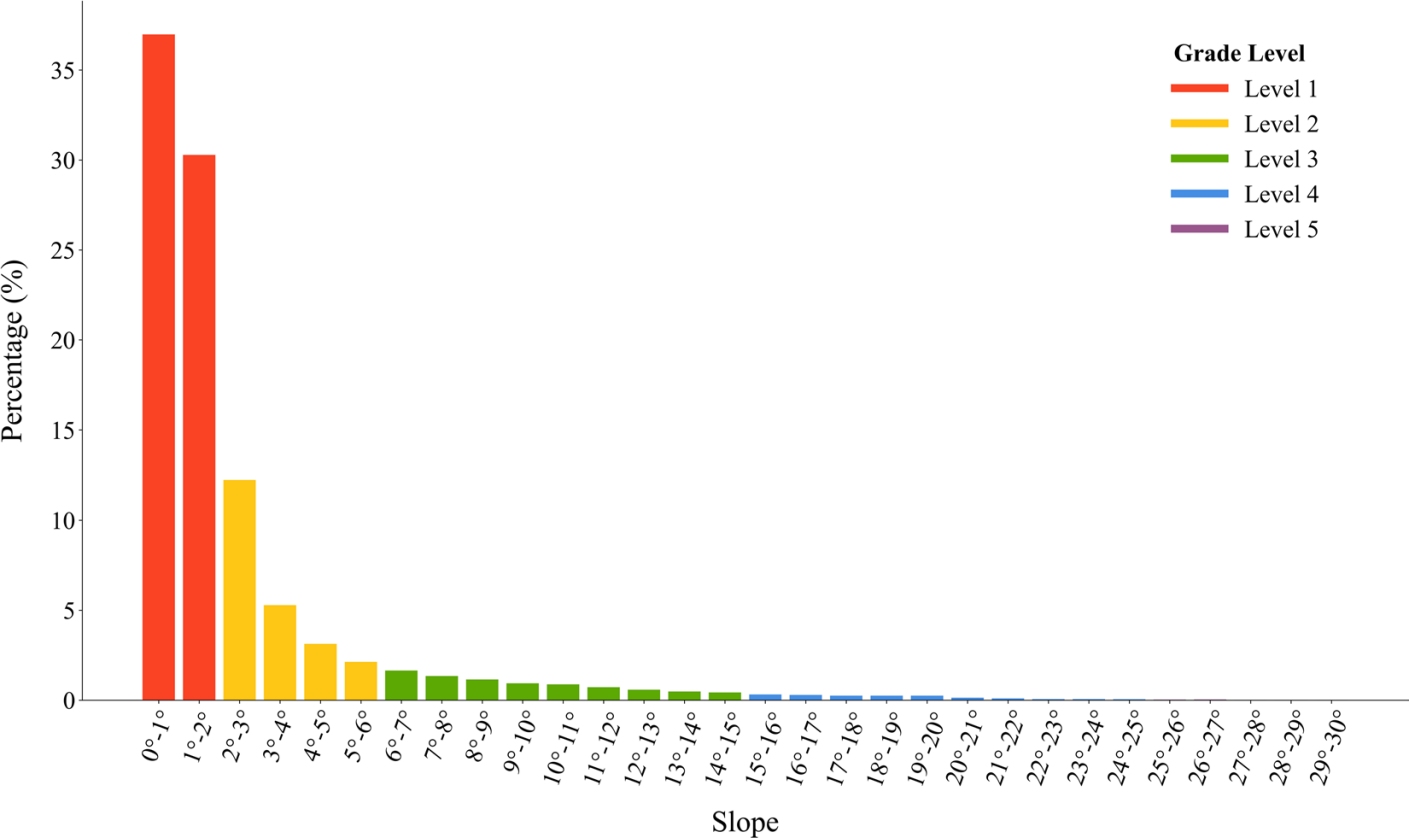
Histogram of slope for PV power stations. Most PV power stations lie in slope under 6°.

Figure 3 shows that in terms of slope, most of the PV power stations (about 90%) lie on flat ground and gentle slope that with an aspect of less than 6°. There are only very few PV power stations (about 1.9%) lie in regions with a slope of more than 15°. This is understandable since the construction workload would be greatly decreased in those flattened areas. Furthermore, the maintenance of PV power stations would also be easier. However, Fig. 3 indicates that even in slopes that are larger than 15 degrees, PV panels could still be placed successfully through the fixed or automatic rotated support.

**Fig. 4:**
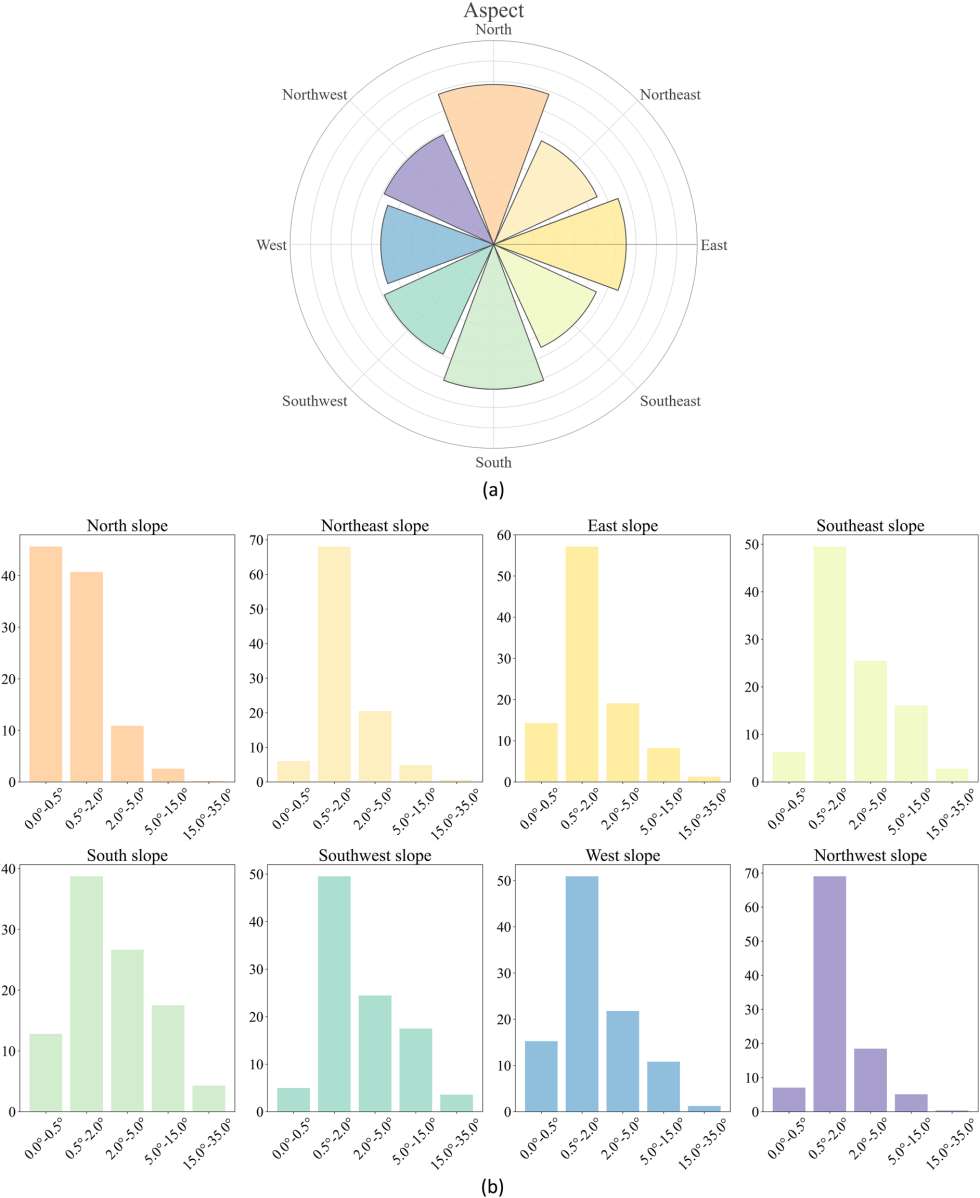
(**a**) spider chart of aspect for PV power stations; (**b**) histogram of slope for PV power stations under each aspect interval.

Figure 4a indicates that China’s PV power stations witness a rather flattened distribution across all the aspects. It should be noted that the aspect here means the orientation of land where PV power station lies, not the orientation of PV panels installation. In fact, the PV panels installation orientation should be close to south to get more solar radiation. However, the aspect of land where PV lies on has little effect on the PV panels installation orientation, since the latter could be adjusted accordingly.

To further investigate this issue, we also calculated the histogram of land slope in each direction (Fig. 4b). It depicts that most of the PV power stations in the northern parts (i.e., north, northeast, and northwest) have a slope of below 5°, i.e., most lying on the flatten ground instead of the nightside of the mountain. Figure 4b also shows that flattened land with small slope is the ideal location to place PV panels, since the installation and maintenance of PV power stations would be easier in such regions.

Besides, after the extraction of both spectral features, textural features, and topographical features, a multi-dimensional feature space has been constructed by feature concatenation. All the PV power stations would be classified in this feature space based on random forest and active learning strategy, which would be elaborated in the next section.

### Modelling

#### Random forest classifier

In this study, a random forest classifier was adopted as the machine learning method to predict PV power stations from multi-dimensional feature space as stated above. The reason why RF was used is according to its effectiveness in modelling multi-linear features^20^. Up to now, RF has been widely used in various remote sensing applications, such as vegetation mapping, water body extraction, etc^30,31^.

Random forest could be viewed as an ensemble classifier that consists of a large number of decision trees^20^, where the classification results would be determined by the vote of each decision tree. Besides, two random sampling processes are performed in RF. The first one is bootstrap sampling, which belongs to an inherent step of RF method. In specific, all the initial training samples now are re-sampled using bootstrap strategy to train each base classifier (i.e., decision tree) of RF. The other is the randomly selection of features used to train each decision tree. Therefore, the above random process makes RF robust to noises and outliers^20^. Meanwhile, the parameterization of RF is rather simple than that of other classifiers such as support vector machine. Only a few parameters are to be tuned including the number of decision trees, the max split number of each tree, and the number of features used to train each tree^20^. All the above parameters have been determined through grid search in this study, where the number of the above three parameters were 200, 10, and 6, respectively.

#### Training details

RF classifier was constructed with the API of GEE, i.e., Classifier.randomForest(). The advantages of using GEE for large-scale mapping are as follows. Firstly, GEE provides the well pre-processed Sentinel-2 data that have been radiometric calibrated, atmospheric corrected, and mosaiced to avoid cloud coverage, which could greatly reduce the burden of remote sensing data downloading and pre-processing^32^. Secondly, GEE also provides the high performance computing service that makes large-scale classification an easy task. According to the above merits, GEE has been popular in various remote sensing applications, such as aquaculture ponds mapping^32–34^, crop mapping^35–37^, etc.

Besides, because the study area is the entire China, the intuitive approach for PV power station mapping is to train a single RF classifier for the entire China instead of partition modelling. However, since the PV power stations (i.e., positive samples) and the surroundings (i.e., negative samples) have high variations in appearance among different provinces of China, which would lead to a high intra-class difference, making it impossible to train one single RF classifier with high accuracy. Therefore, we trained a series of separate RF classifiers for each province of China to make each RF more sensitive to local training samples. By reducing the intra-class variations through partition modelling, it would yield a more accurate local map hence to increase the entire classification performance. Meanwhile, considering that the model’s spatial generalization ability is also very important, therefore, province is selected as the unit of partition modelling rather than city or county. The importance of model’s generalization ability is that model can learn the common and representative features of PV power stations within a large area, making it possible to train a single classifier to achieve a satisfactory accuracy. So how to define the right and reasonable spatial scale to train a single classifier is a key issue. In this study, the PV power stations in the same province of China may share very similar appearances. Besides, in our previous work of mapping China’s agricultural greenhouses^38^, we also selected province as the basic unit and trained a series of RF classifiers. Although city or county could be used as the unit of partition modelling, the workload for training models would increase dramatically. Therefore, training a single RF for each province of China is a trade-off between model’s accuracy, generalization ability and also workload for model training.

Besides, as for some provinces that are too big (including Xinjiang, Inner Mongolia, and Tibet), partition modelling was also performed to split these provinces into several subregions. The reason behind is that the intra-class differences might still be huge for a large province, which would increase the learning difficulty of RF classifier and degrade the classification accuracy. Therefore, subdivision is used to further reduce the intra-class variations and increase the fitting capability of RF to local samples. The practice of subdivision may lower model’s generalization capability, however, for classic machine learning method such as RF, it is difficult to take into account both generalization capability and local sensitivity. Under this circumstance, subdivision of provinces that are too big is also a trade-off between model’s accuracy and generalization ability.

As for the selection of training samples, a stratified sampling method^20^ was used to collect both PV and non-PV samples independently in every provincial unit of China. In specific, before visual inspection from remote sensing images, we performed a detailed search for as many as possible the sites of China’s PV power stations from both relevant research reports, academic papers and news reports. We have also added the PV power station sites derived from our previous field surveys. Afterwards, based on these PV power station sites, we have selected training samples using visual inspection from both Sentinel-2 data and very high resolution Google Earth images. Although we have tried our best to obtain the sites of all the PV power stations of China, it should be noted the above sites still could not cover every PV site of China. To compensate for this, we have also spent lots of time in visual inspection of remote sensing images to collect as many PV samples as possible. Even in those provinces that witness very few PV power stations such as Sichuan, Chongqing, Beijing and Shanghai, we still performed the above process to select both PV and non-PV samples. Another issue is that the selection of non-PV samples is also very important since it would assist in the reduction of false positives (i.e., non-PV predicted as PV) in the classification map. After the labelling process, a total of 320,000 PV samples and 320,000 non-PV samples were collected. Meanwhile, considering that non-PV samples consist of several land covers, therefore, we collect these negative samples from finer categories including forest, grassland, farmland, water, bare land, and impervious surface.

#### Active-learning for improving classification performance

**Fig. 5:**
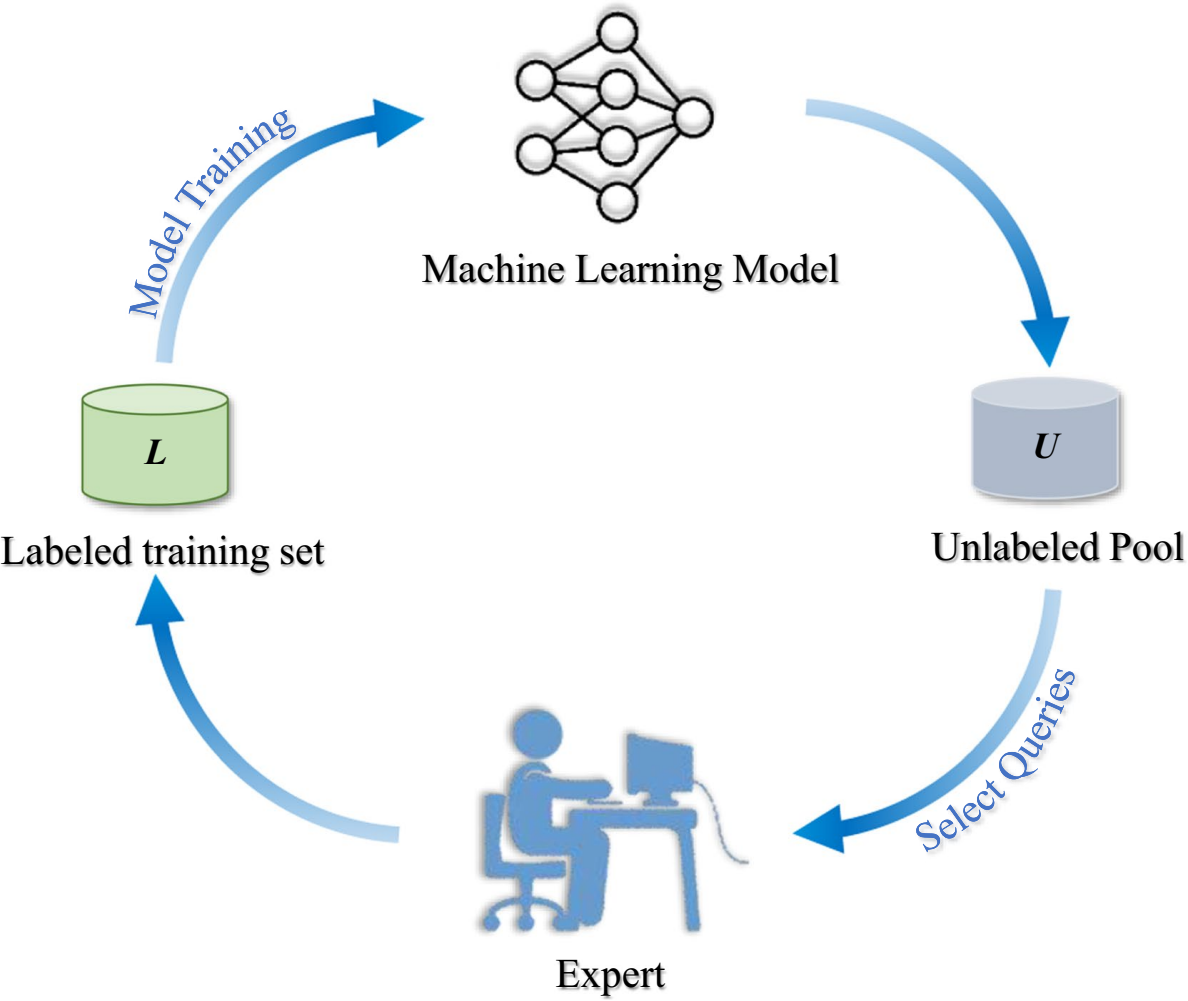
Active learning for re-selecting unlabelled data.

During the mapping process, it would be difficult to produce an accurate map with automatic classification once and only once. In this situation, we resorted to active learning^39^ with a coarse-to-fine strategy to improve the mapping performance iteratively. Actually, active learning originated from machine learning field^39^, which has been a better choice in remote sensing classification under limited labelled samples. The core idea of active learning is described as follows. Firstly, train a classifier with initial labelled data, then use the trained classifier to predict the entire dataset. Afterwards, the wrongly predicted unlabelled data would be sent to experts for further annotation. Then, the classifier would be re-trained with both the initial and the newly labelled data to avoid potential prediction mistakes. The above process would be run in an iterative way until a satisfactory classification result would be achieved (Fig. 5).

In this study, we combined the active learning strategy with RF classifier to refine the PV power station mapping results (Fig. 5). The wrongly predicted pixels were carefully selected and then re-labelled to re-train the RF model. We mainly focused the following two kinds of prediction errors, including the missed PV pixels and these false positive ones to increase the robustness of RF on these typical errors. The above procedure would perform iteratively until the accuracy of PV classification could be satisfied.

Indeed, active learning strategy is a trade-off between automatic machine learning and expert interpretation, aiming to achieve a satisfactory classification accuracy with as less expert interpretation as possible^39^. Meanwhile, the limiting case of expert interpretation is the total or overall vectorization or delineation of each PV power station, however, this would bring in a huge workload for annotators or experts. And the limiting case of automatic machine learning is to train the classifier only once with the initial training samples. Nonetheless, this would not always obtain a good classification map since the initial samples may have a low representativeness. On the other hand, active learning is just a trade-off to increase the accuracy of automatic machine learning at the expense of extra work of human interpretation. With the help of newly added samples, the machine learning classification results would be improved in a coarse-to-fine manner. Besides, according to the experiments (Figs. S1, S2 in the Supplementary file) and our previous study^38^, the number of iterations would be about three times to generate a satisfactory classification result. Therefore, the adoption of active learning and experts’ opinions could increase the classification accuracy with only a small cost of extra annotation work, which could be a good option for large-scale mapping applications.

### Post-processing of the classification data

Note that, since pixel-based classification method has been utilized for satellite remote sensing image, the isolated speckle noises are unavoidable^30^, leading to the so-called salt-and-pepper effect^30^ in the classified PV power station map that would degrade the mapping accuracy. After carefully visual inspection of these noises, most of them have a small size with a total of only several pixels, which is far smaller than that of PV power stations. To eliminate these noises, a post-processing method was adopted, where all the connected regions that are less than 9 pixels (3 × 3) would be removed from the classification map. We also used close operation from mathematical morphology to fill the tiny holes of classification results. To better show the performance of the post-processing method, in the Supplementary file (Figs. S3, S4) we show several cases that and justifies that after the post-progressing step, most of the speckle noises (about 90%) are eradicated while the signal to noise ratio (SNR) improves about 2 db.

## Data Records

The national-scale PV power station map^40^ in this study is provided for entire China in 2020 with a fine spatial resolution of 10 meters, which is the highest resolution recorded among all the publicly released PV datasets. The data format is GeoTIFF while the spatial reference is WGS-84. Meanwhile, only two kinds of values are in the PV power station map, where 0 stands for the non-PV regions while 1 represents the PV power stations. In addition, the provided PV dataset could be loaded into GIS software such as ArcGIS and QIS for data visualization and spatial analysis. Besides, we will continue producing China’s PV power station map in other years, and will release these maps in the future. The dataset^40^ of this study is freely available for all users at Science Data Bank (10.57760/sciencedb.o00121.00001).

## Technical Validation

In this section, we will describe the method for technical and accuracy validation of the PV power station map. Firstly, a national-scale testing dataset has been carefully constructed to perform the accuracy assessment of the mapping results. Afterwards, we will show several detailed PV classification maps to qualitatively evaluate the given dataset.

**Fig. 6:**
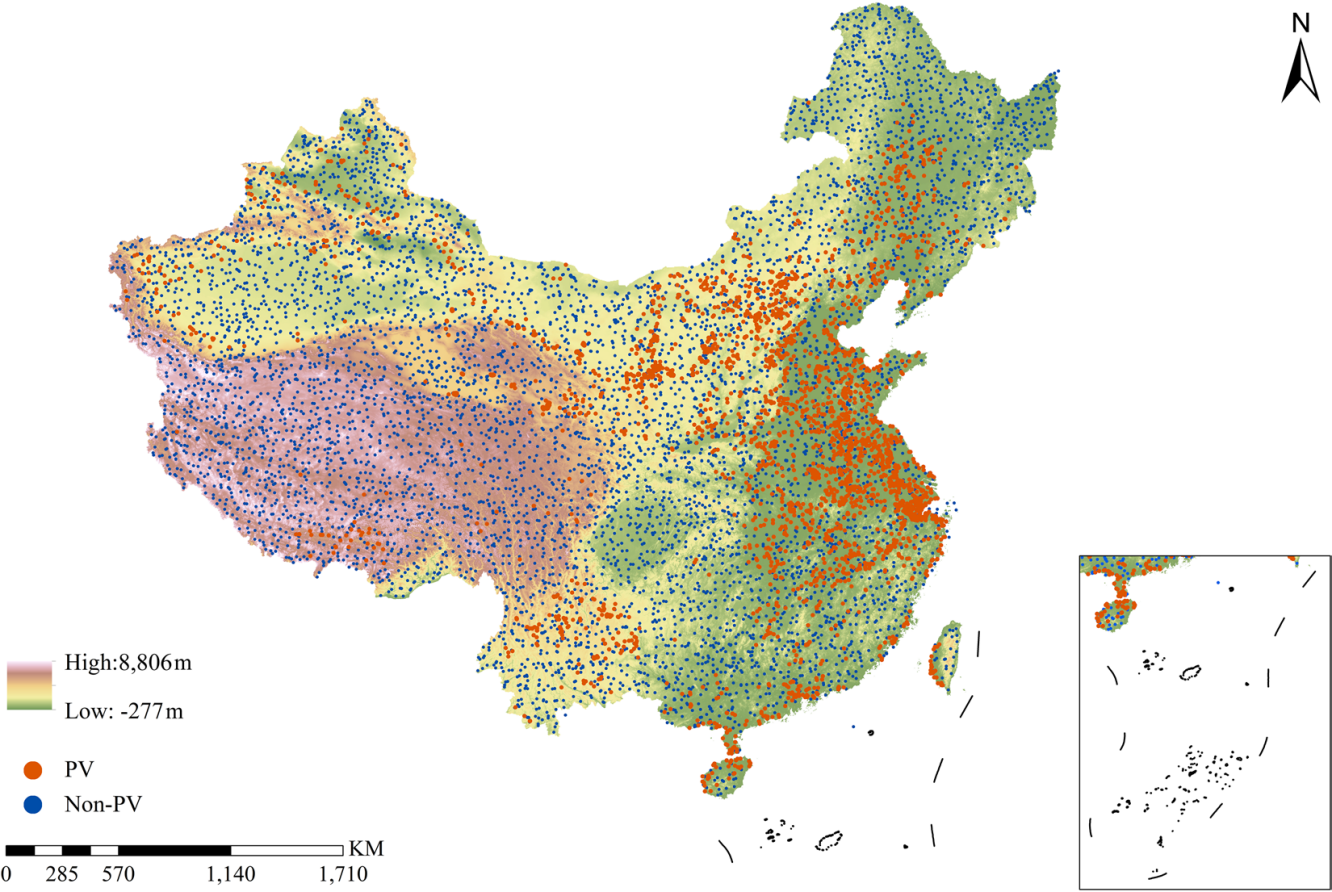
Spatial distribution of testing samples across China.

Specifically, to maintain the repressiveness of the testing dataset spatially, we have selected a total of 10,000 testing samples around China (Fig. 6), including both 5,000 positive samples (i.e., PV) and 5,000 negative samples (non-PV). It should be noted that all these testing samples and the training samples are independent from each other without any spatial intersection. As for the sampling process of testing samples, to maintain spatial uniformity, we divided the entire China into grids with a size of 50 km × 50 km, resulting in a total of 6069 grids nationally, where a stratified sampling strategy was used in all over all the grids to maintain a spatially-balanced sampling process. Since not every grid contain PV stations, we only selected PV samples in certain grids that witness PV panels. Meanwhile, as for negative samples, there are sampled based on all the grids to maintain the spatial representativeness in around China (i.e., about one negative sample per grid).

Both confusion matrix and several accuracy metrics^41,42^ (including recall, precision, F1-score and mIoU) are calculated for both testing samples (Table 1) and training samples (Table S2 in the Supplementary file). It indicates that the training set has shown a very high accuracy with an OA of 98.09%, recall of 0.9777, precision of 0.9851, F1-score of 0.9814 and mIoU of 0.9635. Although the testing set shows a lower accuracy than training set, whose OA, recall, precision, F1-score and mIoU is 89.13%, 0.9014, 0.8836, 0.8057, 0.8924, respectively. Considering that the testing datasets are distributed around the entire China, the classification performance is good enough to understand the spatial patterns of China’s PV stations.

**Table 1:** Confusion matrix derived from testing samples.

Classification results	Testing Data		UA (%)
	PV	Non-PV	
PV	4507	594	88.36
Non-PV	493	4406	89.94
PA (%)	90.14	88.12	

Notes: PA, Producer’s Accuracy; UA, User’s Accuracy.

To better illustrate the PV classification results, we collect several detailed PV maps shown as follows.

**Fig. 7:**
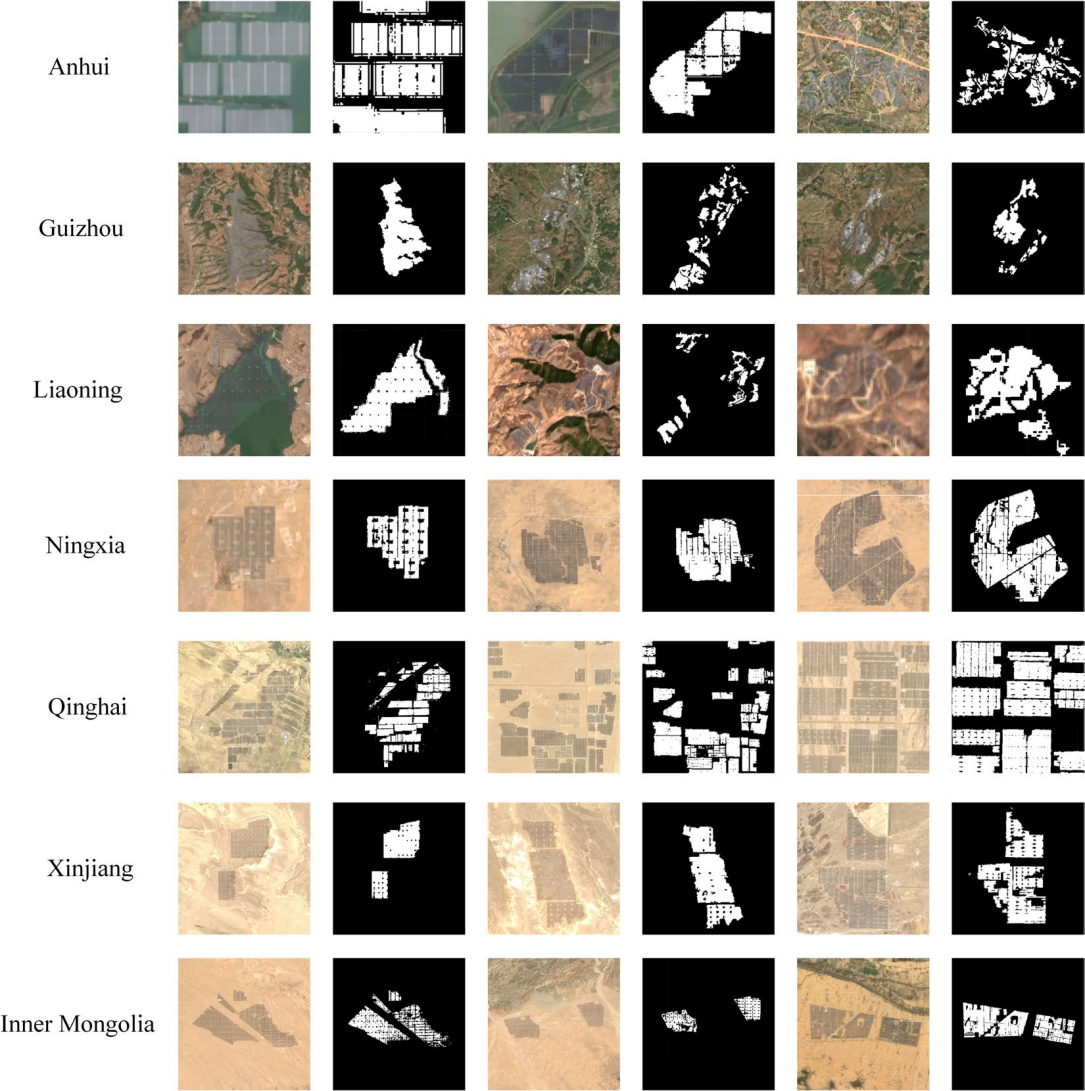
Details of PV power stations map across China.

Figure 7 depicts that PV power stations could be precisely extracted from various backgrounds such as gobi, water bodies, grassland and mountainous regions. Since the 10-m Sentinel-2 imagery was utilized, a detailed boundary of PV power stations could be obtained, which is a merit over Landsat-derived 30-m PV maps. In addition, based on the provided national-scale PV map and the associated Sentinel-2 imagery, it would be easy to obtain numerous image and mask patches to train a deep learning based semantic segmentation model for PV classification in the near future. Therefore, the PV dataset in this study could also be viewed as a benchmark for deep learning based studies.

Figure 7 also shows that some roads and related facilities inside the PV power stations are classified into non-PV, this is mainly because we did not select theses regions as PV samples. On the contrary, if we selected roads and related facilities (mainly power transformation equipment) within PV power stations as positive samples, this would lead to the appearance of more false positives in the final classification map. Other land cover types especially the roads and industrial roof tops may be misclassified into PV power stations. The drawback of this study is that roads and other facilities have not been classified, leading to a risk of underestimating the areas of PV power stations. In fact, the discard of roads and other facilities is actually a trade-off to reduce false positives, which is an inevitable issue in large-scale mapping applications. Besides, although roads and other facilities are not recognized, most of the PV panels are classified correctly, which would also reflect the spatial pattern of China’s PV power stations.

In addition, one merit of random forest is that it could yield the importance of input features. To further validate this merit, we have calculated the variable importance of each province and then obtained the top-10 variables based on the average importance values. Meanwhile, we have also trained a single RF classifier for the entire China using the combination of training samples from each province and then calculated the variable importance (Fig. S5 in the Supplementary file). It indicates that the top-10 important variables are the same for both entire modelling and partition modelling, including NDBI, NDPI, Band-8, Band-2, slope, Band-12, elevation, SAVI, NDVI and Band-4 of Sentinel-2. More specifically, among these ten variables, several variables show very high importance in both partition modelling and entire modelling, including NDBI, NDPI Band-2, slope and Band-12. The reason is that both NDBI and NDPI could increase the separability between PV power stations and buildings. Band-2 is blue band and most of PV panels show the colour of dark blue from the satellite image. Slope is an important parameter that determines the suitable positions of PV power stations. While Band-12 is the key parameter in NDPI, which has also a high importance. Above all, these variables have more impact on the classification of PV power stations, which could be paid more attention in future relevant studies. The only exception is elevation, which has a high average importance and high deviation in partition modelling but has a low value in entire modelling. The reason may be that in partition modelling, the variations of elevation in each province is not that high when compared with the entire China, therefore, the correlation between elevation and PV power stations is higher locally (i.e., at provincial scale) than globally (i.e., at national scale).

Besides, we have taken a series of steps during the remote sensing classification to maintain the data’s accuracy and reliability as follows.

### Quality control of labelled samples

A strict training course has been taken for all the annotators for visual interpretation, which could help them recognize PV power stations under various backgrounds. Both variability and correctness of PV samples are required for each annotator. After the initial labelling procedure, annotators are asked to perform a cross-check step to further refine the sample’s quality.

### Quality control of remote sensing imagery

Firstly, the high-resolution Sentinel-2 data are selected to reduce the mixed pixels hence to reserve as much as the details of PV power stations. Secondly, due to the short revisit time of Sentinel-2, it is easier to generate cloud-free images on GEE, which is of great significance to large-scale remote sensing classification.

### The inclusion of multiple features for classification

To increase the inter-class difference between PV power stations and other LULC categories, both spectral features, texture features and topographical features have been extracted.

### Partition modelling

A difficulty for large-scale remote sensing classification is the huge intra-class variations. To tackle this issue, a partition modelling strategy is utilized to increase the fitting ability of classifiers for the local samples.

### Active learning for coarse-to-fine classification

Active learning is used to select the typical wrongly predicted pixels to be re-labelled. Afterwards, the RF classifier would be re-trained with the refined labelled data to reduce the classification errors iteratively.

### Post-processing of the speckle noises

To further refine the classification results, post-processing step is taken to improve the salt-and-pepper effect.

## Usage Notes

We have released the distribution map of China’s PV power stations in the unit of province. The PV map is in the standard format of GeoTIFF, which could be easily further processed by both GIS software and coding language such as R and Python. The GEE code for generating PV map has also been released for the purpose of reproduction, including the calculation of each feature, random forest classification, etc. Besides, the released code could also provide a useful reference for other large-scale mapping applications such as cropland mapping, flood mapping, etc.

Each pixel in the released PV maps has the area of 100 m^2^ (10 m × 10 m). Meanwhile, because we have used post-processing method to filter the speckle noises that are less than 3 × 3 pixels (i.e., 900 m^2^), therefore, the smallest PV station that can be mapped is 900 m^2^. Since we are mainly focused on the ground mounted PV stations, whose areas are much more than 900 m^2^ while even the small PV stations in China are about 30,000 m^2^. Therefore, the post-processing step would not lead to the possibility of missing out these small ground-mounted PV stations.

**Fig. 8:**
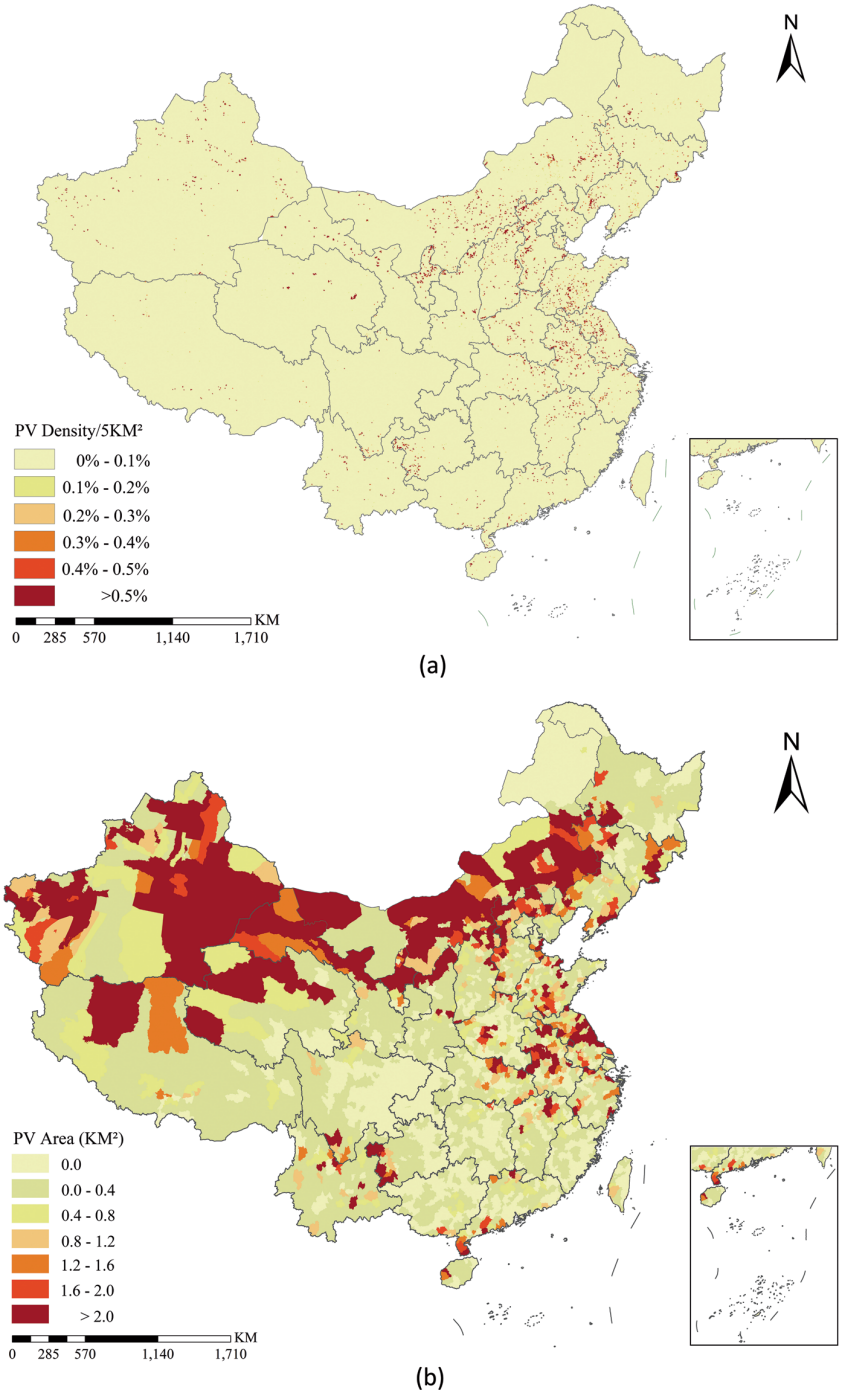
(**a**) PV power stations density map across China; (**b**) PV power stations area map for each county of China.

Based on the provided PV dataset, it would be easy to illustrate where these PV power stations lie across entire China. Considering that the classified PV power stations only account for a very small ratio of China, if we directly put these PV pixels on a national-scale map, it would be difficult to show their spatial distribution patterns. To tackle this issue, we first split entire China into a series of grids with a size of 5 km × 5 km, then calculated the percentage of PV pixels and finally put these PV area ratios on a national-scale map (Fig. 8a).

Figure 8a illustrates that most of PV power stations lie in the northern part of China, especially in northwest and northeast China. Interestingly, a large number of PV power stations lie along the Great Wall (including the northern parts of Hebei, Shanxi, Shaanxi, Ningxia and Gansu Province) and the Silk Road (mainly refers to Gansu and Xinjiang Province). Meanwhile, in eastern China, PV power stations mainly locate in Anhui, Jiangsu, Shandong, Henan, Hubei and Jiangxi Province, while in southwestern China, Guizhou, Yunnan and Sichuan witnessed the most PV power stations.

Apart from the grid map, we also calculated the areas of PV power stations in each county of China and put these data on map (Fig. 8b). Compared with the grid map, county-level PV map could provide the panel data of PV power stations of each county, which could facilitate in-depth analysis with socio-economic data, since most socio-economic data in China are in the units of county. In this context, Fig. 8b could also provide policy implications for decision makers.

**Fig. 9:**
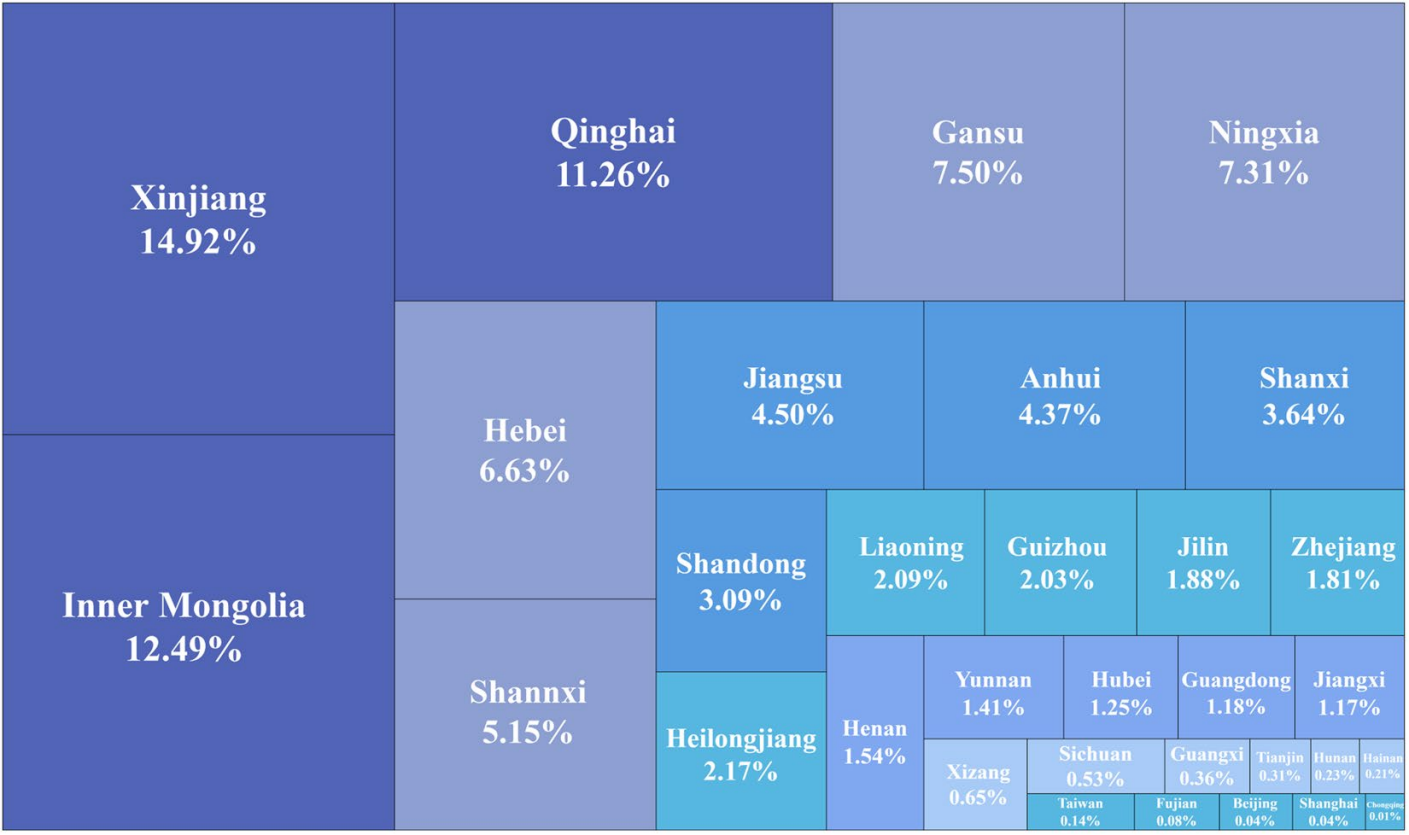
Areas of PV power stations for each province of China.

Finally, based on the provided national-scale PV map, we also calculated the area ratio of each province (Fig. 9). According to our dataset, China has a total of 2467.7 km^2^ ground-mounted PV power stations in 2020. The top three largest provinces refer to Xinjiang, Inner Mongolia and Qinghai, whose PV area ratio are 14.92%, 12.49% and 11.26%, respectively, with a total of nearly 40% of all the PV power stations of China. Among the rest provinces, both Gansu, Ningxia, Hebei and Shaanxi witness a PV area ratio above 5%, which contributes to another 20% of China’s PV power stations in total. It should also be noted that with the rapid development of China’s PV industry, increasingly more eastern provinces built large-scale PV power stations, including Jiangsu, Anhui and Shandong Province.

The above analysis indicates that China’s PV power stations mainly locate in three regions, including the northern, eastern and southwestern parts of China. The driving forces behind are analysed as follows. First, as for Northern China (especially Xinjiang, Qinghai, Gansu, Ningxia and western part of Inner Mongolia), it has a typical arid and semi-arid climate with very few cloudy and rainy weathers, leading to a long effective sunshine duration. Besides, these regions have large-scale gobi and desert with a rather flat terrain, which is suitable for placing PV power stations. Second, as for Eastern China (especially Shandong, Jiangsu and Zhejiang), it has developed industries which contribute to more carbon emissions than Western China. Therefore, to response to China’s Carbon Peak and Carbon Neutrality strategy, provinces in Eastern China have also constructed PV power stations, aiming to produce more clean energy and reduce the consumption of fossil fuel. Finally, As for Southwestern China (especially the border regions for Sichuan, Guizhou and Yunnan), the placement of PV power stations is for the purpose of reducing poverty. Local residents could be benefited from the land subsidy, making it possible to maintain both clean solar energy production and poverty alleviation, both of which are highly valued by China’s central government.

Besides, the released dataset is mainly focused on ground-mounted PV power stations of China and not yet consider distributed PV stations such as rooftop PV systems. Two reasons may account for this. Firstly, rooftop PVs are more scattered and in small size. Considering that the spatial resolution of Sentinel-2 data is 10 meters, and rooftop PVs do not manifest themselves obviously under this resolution, which will increase the difficulty of extracting the rooftop PVs. Secondly, the grounded-mounted PV power stations almost cover more than 90% of the total PV capacity in China, therefore, even without distributed PV systems, the released dataset could also locate most of the PV industries in China. Moreover, in future studies, we will consider utilizing very high resolution data (i.e., WorldView-3/4) and deep learning models to produce rooftop PV data.

To sum up, we provide a 10-m map for China’s PV power stations to provide reference data to understand the spatial pattern of China’s PV industry. The dataset could also be used for other applications such as prediction of PV’s generating capacity and site selection for newly built PV power stations. Besides, the detailed PV map could also support for policy making of China’s clean energy and provide useful data for studies such as land use and land cover change.

### Supplementary information

Supplementary_Information

## Data Availability

The GEE code for PV power stations classification based on Sentinel-2 imagery and DEM data is available at https://github.com/MrSuperNiu/PV_ScientificData_Classification_Code. The code is written in JavaScript, including all the mentioned steps in this paper, including feature calculation, random forest training, etc.
