# Supplementary material for: A 10-m national-scale map of ground-mounted photovoltaic power stations in China of 2020
Source: Sci Data. 2024 Feb 13;11:198. doi: 10.1038/s41597-024-02994-x (PMC10864270; doi:10.1038/s41597-024-02994-x)
Supplement: Supplementary file 1 — Supplementary_Information [file 41597_2024_2994_MOESM1_ESM.docx]

**Supplementary Contents**

Table S1.1

Table S2.3

Fig. S14

Fig. S24

Fig. S35

Fig. S45

Fig. S56

**Table S1**. Terminologies and definitions.

| **Abbr.** | **Full name** | **Definition** | **Formula** |
| --- | --- | --- | --- |
| **DEM** | Digital Elevation Model | Digital Elevation Model (DEM) depicts the Earth's bare ground topography, omitting trees, buildings, and other surface objects. These models are generated using various data sources. | -- |
| **NIR** | Near Infrared | The near infrared band of satellite image. | -- |
| **SWIR** | Short Wave Infrared | The short wave infrared band of satellite image. | -- |
| **NDVI** | Normalized Difference Vegetation Index | The normalized difference vegetation index (NDVI) is a commonly employed method to assess the health and density of vegetation with sensor data. |  |
| **SAVI** | Soil-Adjusted Vegetation Index | The Soil-Adjusted Vegetation Index (SAVI) method is a vegetation index designed to mitigate the impact of soil brightness by incorporating a soil-brightness correction factor. |  |
| **MNDWI** | Modified Normalized Difference Water Index | The Modified Normalized Difference Water Index (MNDWI) is method to distinguish between water and urban areas in satellite imagery. |  |
| **NDBI** | Normalized Difference Building Index | The Normalized Difference Built-up Index (NDBI) is a ratio that characterizes the density of built-up structures in a geographical region. |  |
| **NDPI** | Normalized Difference Photovoltaic Index | The Normalized Difference Photovoltaic Index (NDPI) is a method utilized to identify photovoltaic features within a provided satellite image. |  |
| **GLCM** | Gray Level Co-occurrence Matrix | The GLCM function assesses the texture of an image by determining the frequency with which a pair of pixels, defined by specific values and spatial relationships, appear in the image. | -- |
| **ASM** | Angular Second Moment | The Angular Second Moment method is used to calculate the mean value of the gray scale distribution and the thickness of the texture of the image, also known as the energy value, the larger the value, the more homogeneous or regular the pixels are in the window. |  |
| **CONTRAST** | Contrast | The Contrast method is used to calculate the degree of contrast in the gray scale of an image, characterizing its texture shotgun and clarity, and the larger its value, the more pixels of high contrast within that window, the clearer the image. |  |
| **CORR** | Correlation | The Correlation method is used to measure the similarity of the elements of the grayscale symbiotic matrix in the row or column direction, which could reflect the local grayscale correlation of this window, the larger the value, the smaller the difference of its matrix pixels. |  |
| **VAR** | Variance | The Variation method is used to calculate the spatial non- homogeneity of the image, the larger the value, the brighter the image display. |  |
| **IDM** | Inverse Difference Moment | The Inverse Difference Moment method is used to calculate the clarity and regularity of the image texture. |  |
| **MEA** | Mean | Sum Average is used to reflect the degree of regularity of the whole image texture, the larger the value, the more regular in the whole image texture. |  |
| **ENT** | Entropy | The Entropy method is used to measure the overall image clutter and it also represents the amount of information, the larger the value, the more cluttered the image is. |  |
| **DISS** | Dissimilarity | The Dissimilarity method is used to measure the degree of contrast or spatial heterogeneity of the individual pixel gray values of an image, the larger the value, the more prominent the image texture information. |  |

**Table S2**. Confusion matrix derived from training samples.

|  | Training Data | |  |
| --- | --- | --- | --- |
| Classification results | PV | Non-PV | UA (%) |
| PV | 165970 | 2506 | 98.51 |
| Non-PV | 3780 | 157486 | 97.66 |
| PA (%) | 97.77 | 98.43 |  |

Notes: PA, Producer’s Accuracy; UA, User’s Accuracy.


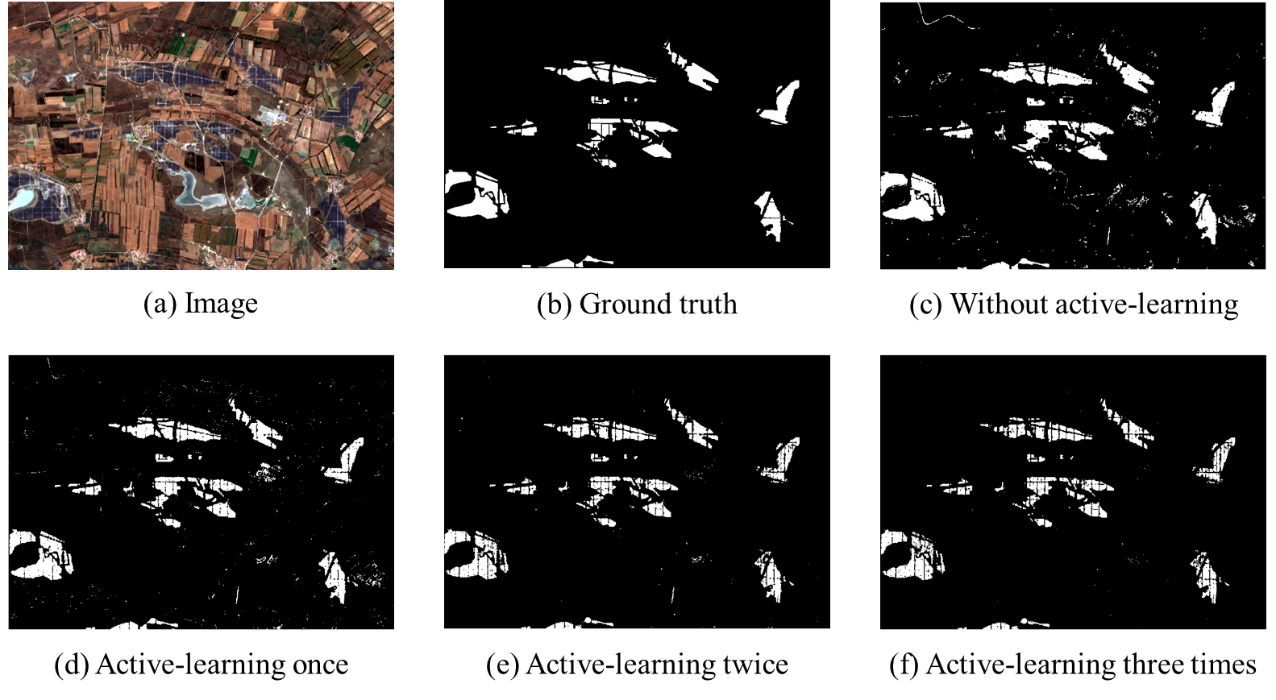


**Fig. S1** Case-1 for active learning.


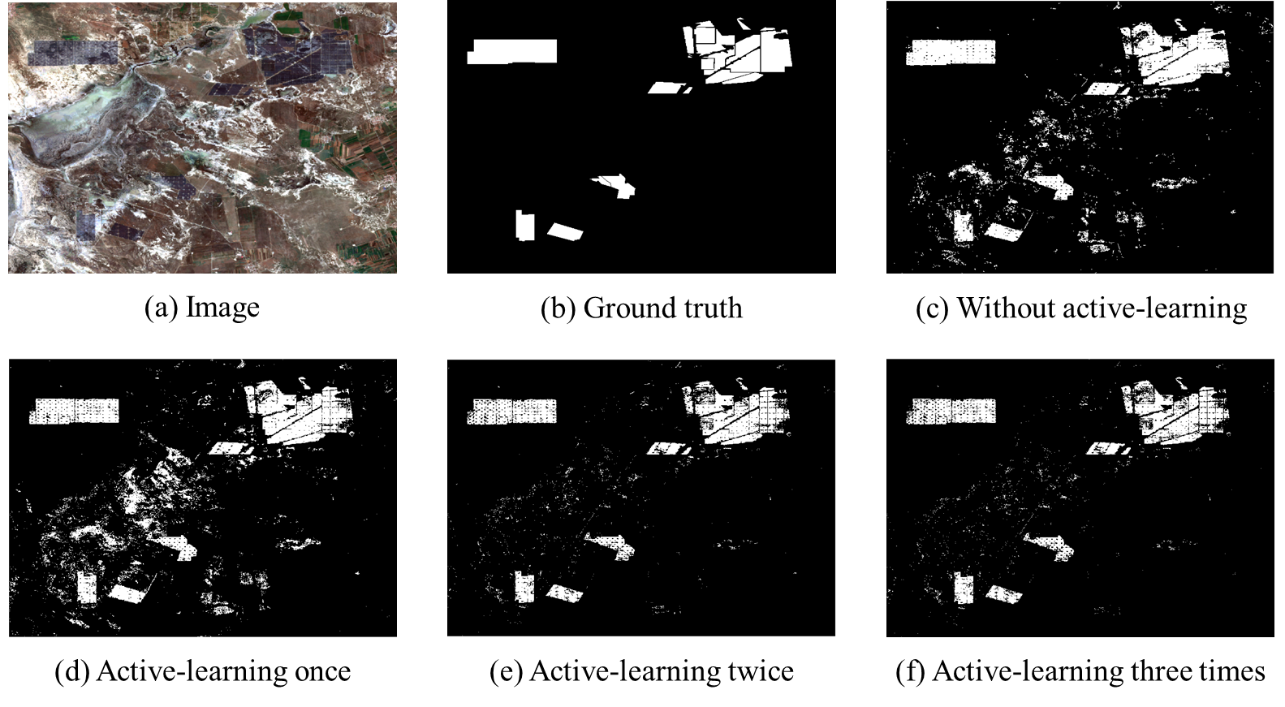


**Fig. S2** Case-2 for active learning.


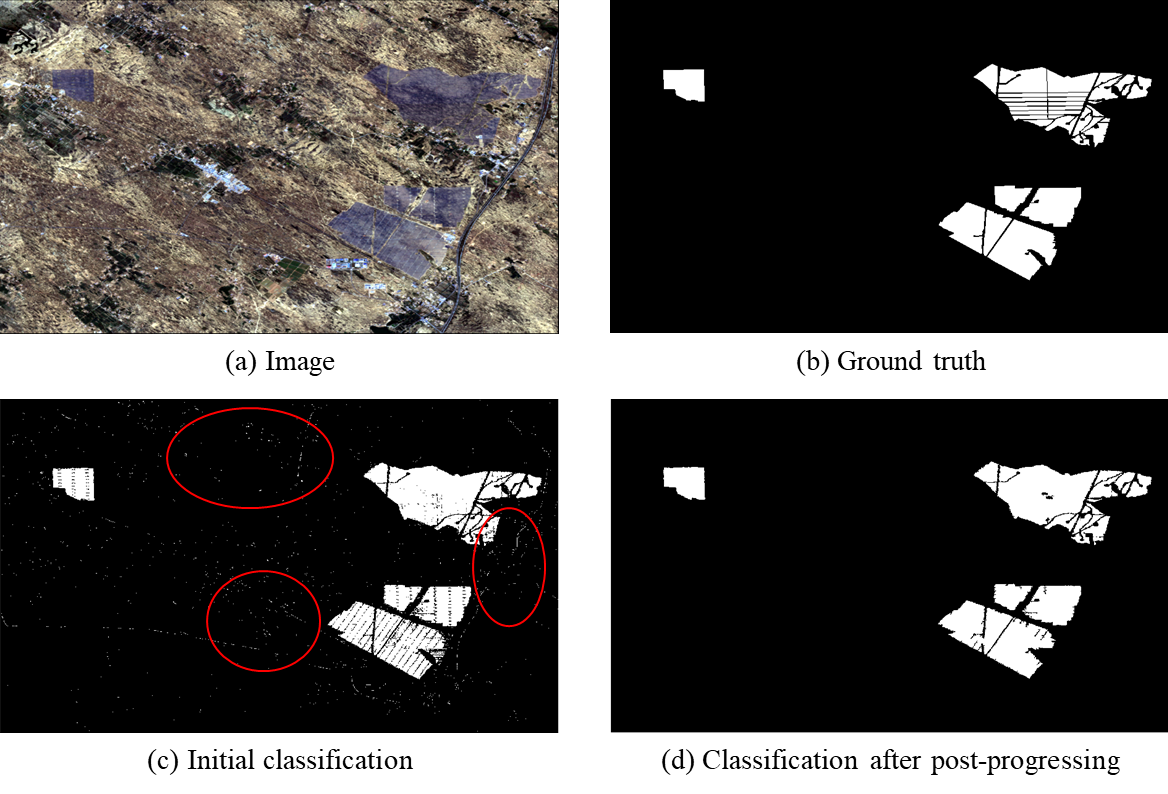


**Fig. S3** Case-1 for PV mapping results before and after post-progressing. Note, red circle denotes the speckle noises.


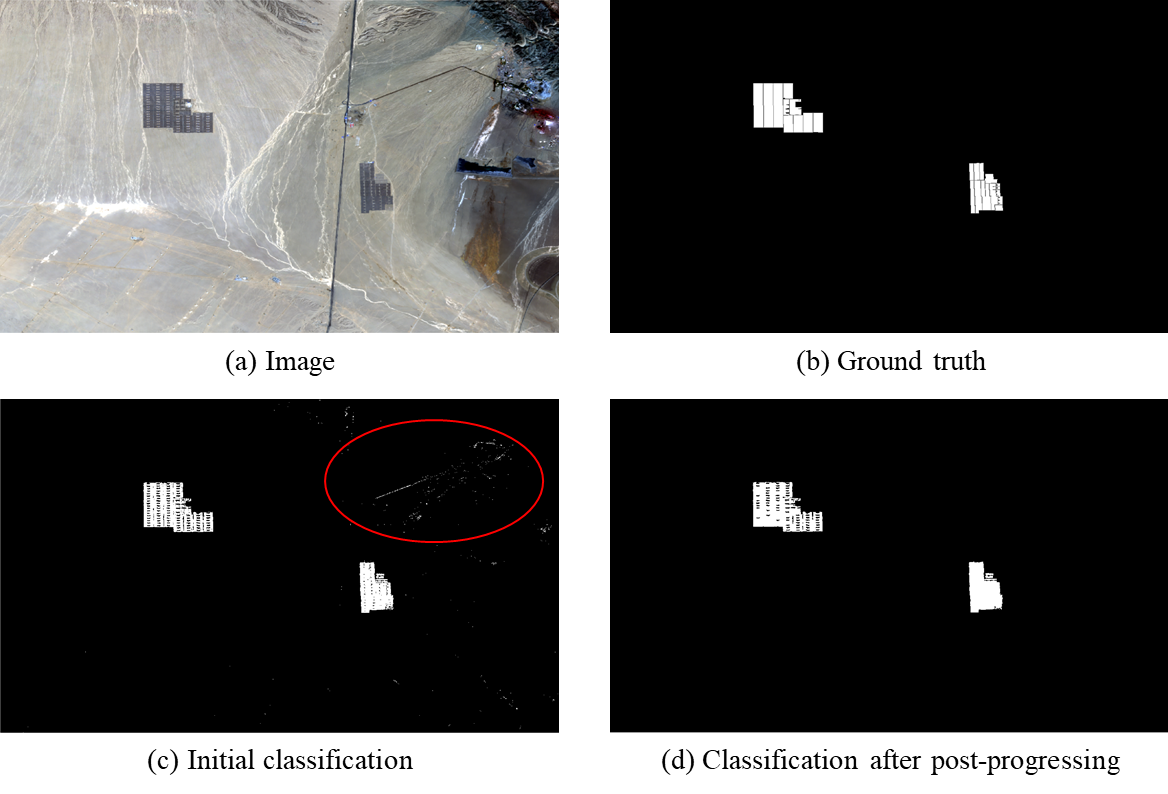


**Fig. S4** Case-2 for PV mapping results before and after post-progressing. Note, red circle denotes the speckle noises.


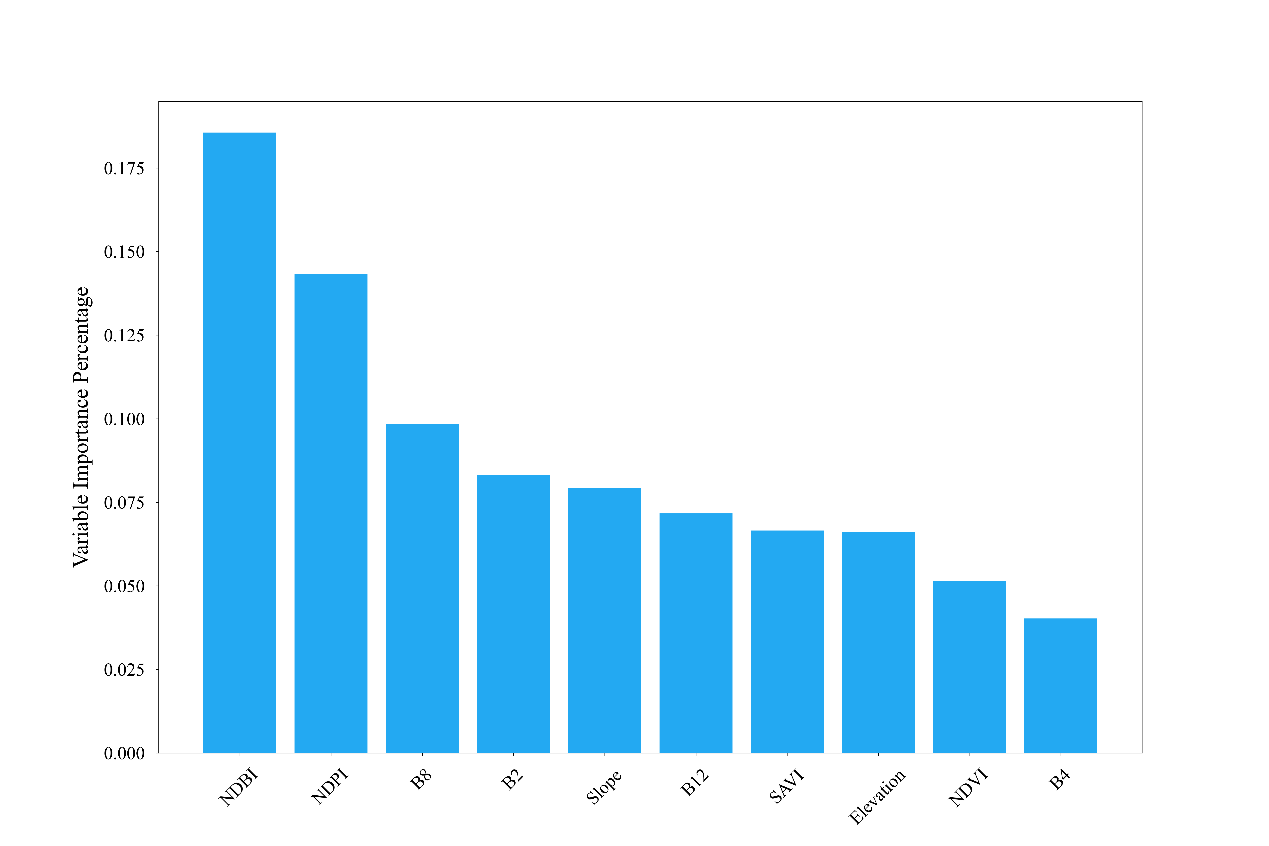


(a) Variable importance obtained from a single RF classifier for the entire China


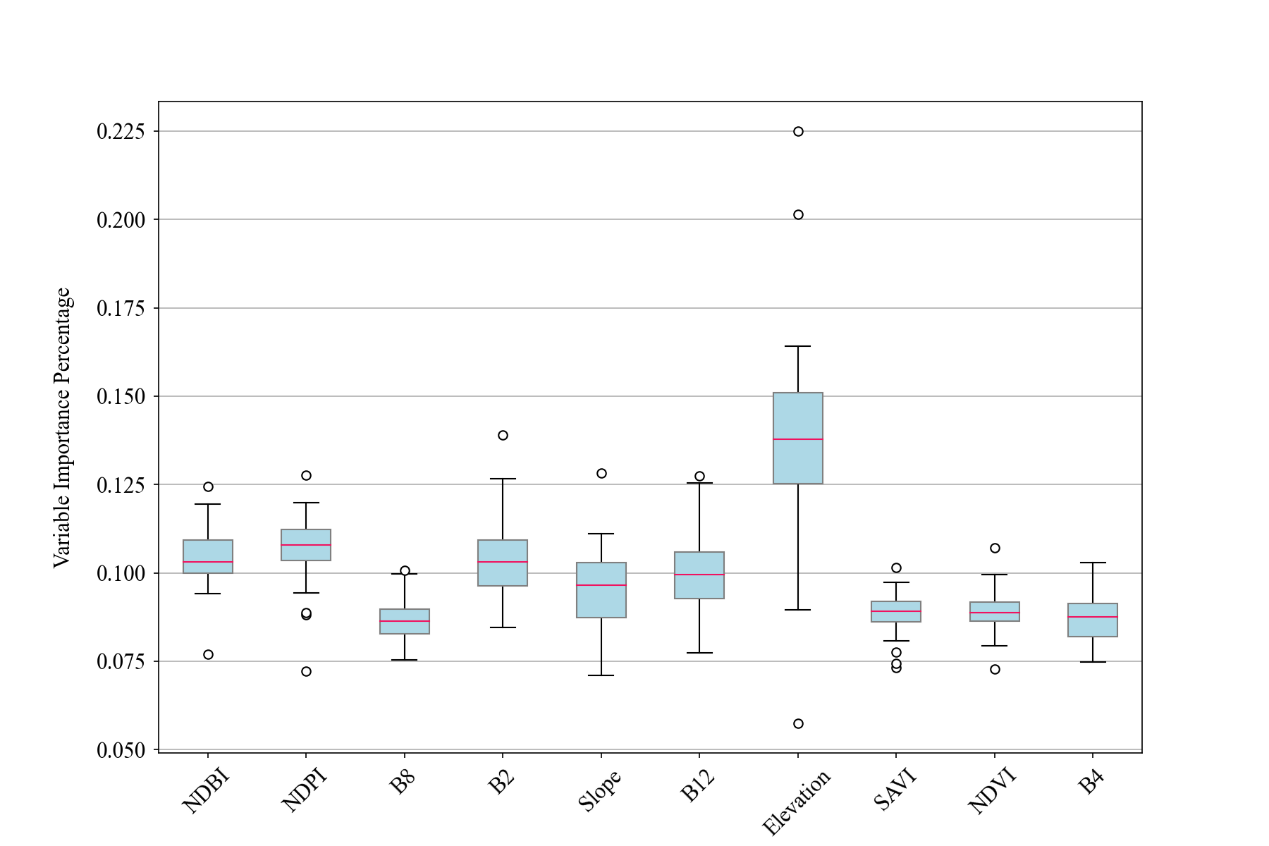


(b) Variable importance obtained from a series RF classifier for partition modelling

**Fig. S5** Variable importance for both entire modelling and partition modelling.
